# Production and Characterization of Sumac PlantCrystals: Influence of High-Pressure Homogenization on Antioxidant Activity of Sumac (*Rhus coriaria* L.)

**DOI:** 10.3390/plants10061051

**Published:** 2021-05-23

**Authors:** Abraham M. Abraham, Camilo Quintero, Luis Carrillo-Hormaza, Edison Osorio, Cornelia M. Keck

**Affiliations:** 1 Department of Pharmaceutics and Biopharmaceutics, Philipps-Universität Marburg, Robert-Koch-Str. 4, 35037 Marburg, Germany; abraham.abraham@pharmazie.uni-marburg.de; 2 Grupo de Investigación en Sustancias Bioactivas, Facultad de Ciencias Farmacéuticas y de los Alimentos, Universidad de Antioquia UdeA, Calle 70 No. 52-21, 50010 Medellín, Colombia; camiloa.quintero@udea.edu.co (C.Q.); luis.carrillo@udea.edu.co (L.C.-H.); edison.osorio@udea.edu.co (E.O.)

**Keywords:** plants, *Rhus coriaria L*., *Anacardiaceae*, sumac, PlantCrystals, extracts, high-pressure homogenization, nanomilling, antioxidant capacity, green extraction

## Abstract

Oxidative stress diseases are usually treated or prevented by using antioxidants from natural or artificial sources. However, as a sustainable source of phytochemicals, plants got a renewed interest in obtaining their active agents using green extraction technologies, i.e., sustainable extraction techniques that reduce energy consumption, use renewable sources and result in less post-extraction wastes. The high-pressure homogenization (HPH) technique was introduced into the food industry since it was invented in 1900 to homogenize milk and later to produce fruit juices with a longer shelf-life without preservatives. Recently, HPH was introduced as an eco-friendly method to nanomill plants for improved extraction efficacy without using organic solvents. In this study, sumac was used as an antioxidants-rich spice model to investigate the effects of HPH on its antioxidant capacity (AOC). Sumac was rendered into PlantCrystals by using HPH. Particle size characterization proved the presence of submicron-sized particles (about 750 nm). Thus, HPH was able to produce sumac PlantCrystals and increased the AOC of bulk sumac by more than 650% according to the ORAC (oxygen radical absorbance capacity) assay. The polyphenol and flavonoid contents showed higher values after HPH. Interestingly, the DPPH (1,1-diphenyl-2-picrylhydrazyl) assay also showed a well improved AOC (similar to ascorbic acid) after HPH. In fact, in this study, the PlantCrystal-technology was demonstrated to cause an efficient cell rupture of the sumac plant cells. This caused an efficient release of antioxidants and resulted in sumac PlantCrystals with a 6.5-fold higher antioxidant capacity when compared to non-processed sumac bulk material.

## 1. Introduction

Oxidative stress diseases are considered as one of the serious disorders caused by the imbalance between the free radicals and the antioxidants in the human body [1]. This imbalance is due to the reduced biological system’s ability to detoxify the reactive intermediates or repair the oxidative stress resulted damage. Reactive oxygen and nitrogen species (ROS and RNS, collectively RONS) are responsible for this damage. RONS are reactive derivatives of cellular oxidation processes characterized by the presence of unpaired electrons in their outer shells (i.e., free radicals). Antioxidants are a counterbalance to the reactivity of RONS that work by direct quenching or scavenging of RONS or indirect, by reducing oxidized substrates [2] and stimulating the transcription of other antioxidant systems [3]. Many antioxidants, from natural or artificial sources, can be used to counteract RONS. Thus—due to their antioxidative properties—there is an increased and renewed interest in using plant secondary metabolites, not only in pharmaceuticals but also in other products, i.e., for healthcare and cosmetics.

Due to the increased demand of plant extracts and the limited availability of natural plants, there is not only a great interest in obtaining plant extracts with high biological activity but also by using efficient and green extraction technologies at the same time [4,5,6]. The term “green extraction” was proposed by Chemat et al. and is defined as “sustainable extraction technique” that reduces energy consumption, uses renewable natural products and results in less post-extraction wastes [7]. Furthermore, these green extraction techniques should result in a complete extraction processes that can be performed in minutes instead of hours. In addition, the process should be highly reproducible and reduce the use of organic solvents [7,8]. Hence, today not only effective plant extracts but also eco-friendly methods need to be developed to introduce plants’ phytochemicals.

Plants—as a source of bioactive components—are frequently consumed in nutrition and have been used as traditional medicines for generations, where they are still used in form of classical extracts such as infusion, decoction or maceration in aqueous media [9,10,11]. To use their nonsoluble active compounds in pharmaceutical and personal-care products, their extracts are usually prepared using organic solvents due to their constituents’ low solubility in aqueous media [12]. These methods require using organic solvents and a huge amount of plant starting materials (with the resources for growing, harvesting, transportation requirements, etc.). Therefore, environmentally benign processes that improve sustainability and the eco-friendly production of such products are of high importance and should be developed in an efficient and low-cost manner.

**Figure 1:**
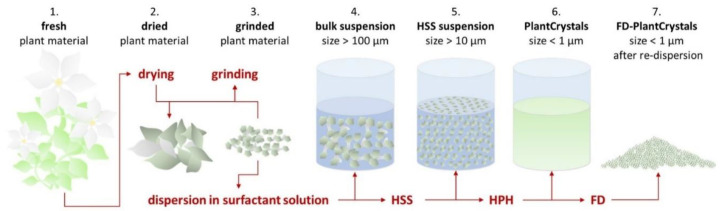
Scheme of PlantCrystals production. HSS = high speed stirring using rotor-stator homogenizer, HPH = high pressure homogenization at 1500 bar using a piston-gap homogenizer, FD = freeze drying. Modified after [24].

**Figure 2:**
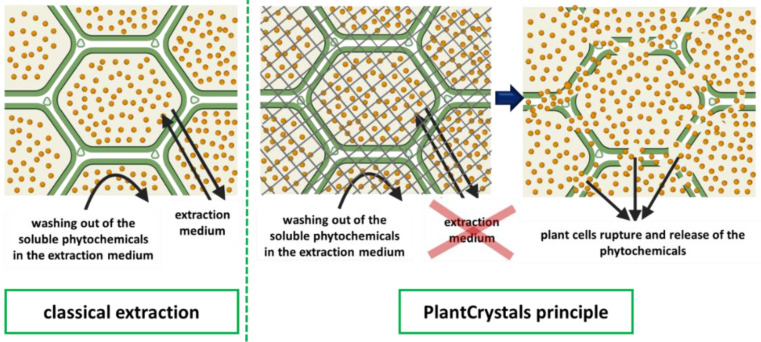
Comparison between the principle of the classical extraction process and PlantCrystal-technology. The green lines represent the plant cells and the yellow round particles represent the phytochemicals inside the plant cells. The grey net (in the middle image) represents the forces applied during HPH for plant cells rupture.

One of these recently used methods to achieve this is high-pressure homogenization (HPH) to nanosize plant material and/or their wastes [13,14,15]. HPH technique was introduced into food industry since it was invented by Auguste Gualin in 1900 [16]. It is used for juices to reduce the microbial load, increase juice uniformity and reduce serum separation, while preserving the quality of the fresh juices [16,17,18]. However, one of the most recent applications of HPH is to nanomill medicinal plants to produce PlantCrystals with improved health beneficial effects [13,14,19]. PlantCrystals are composed of 100% milled plants or parts of plants and possess sizes in the submicron range (Figure 1). The technology uses the whole plant material and destroys all plant cells, which ensures a complete release of its phytochemicals (Figure 2). The novel approach can therefore improve the bio-efficacy of medicinal plants [13,20]. It is environmentally friendly because the use of organic solvents is not required, no organic waste is produced and less plant material is required [13,14,15]. The PlantCrystals can be obtained by several wet milling procedures, like bead milling, high-speed stirring (HSS), high-pressure homogenization (HPH) or combinations of these methods [13]. This results in aqueous PlantCrystal suspensions that can be freeze dried to increase their microbial stability (Figure 1).

The above-mentioned methods for the production of PlantCrystals are already well-established in the pharmaceutical industry, where they are exploited for the production of drug nanocrystals [21,22,23]. Hence, the PlantCrystal-technology can be regarded to be a cost-effective, eco-friendly and sustainable production method that can also be used for large scale production in a GMP environment. With the above-mentioned features, the PlantCrystal-technology can be considered to be a universal formulation approach for herbal medicinal pharmaceuticals and personal-care products. However, recent studies also demonstrated that nano-milling and/or subsequent freeze drying—due to the production of heat and/or the incorporation of oxygen—can destroy chemically labile compounds. The freeze drying can also affect the physical stability of the formulations and can cause agglomeration and with this a loss in bioactivity of the PlantCrystals. The hazard of chemical degradation and physical instability depends on the production method, the production parameters used and—most importantly—on the type of plant material itself [13,14,20].

Despite various studies that prove the versatility of the PlantCrystal-technology, a systematic understanding of most suitable process parameters and production methods for the different types of plant material (leaves, flowers, roots, etc.) are not yet available. Therefore, the PlantCrystal-process needs to be developed and optimized for each plant material individually. The aim of this study was to investigate if the bioactivity of sumac can be increased by transferring sumac fruits into sumac PlantCrytsals.

**Figure 3:**
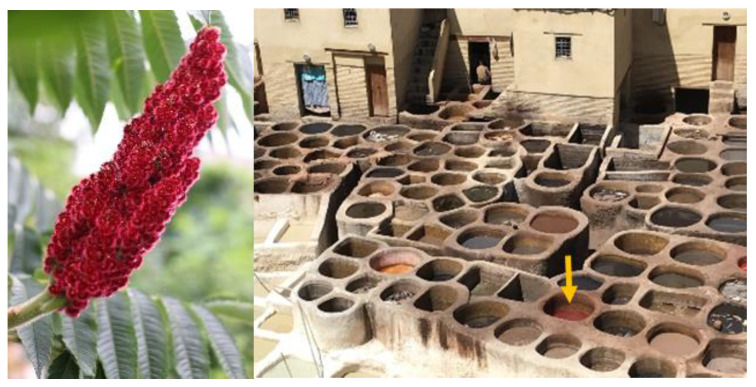
Sumac (*Rhus coriaria L.*) plant photo was taken in Marburg in July 2020 (left) and traditional tanneries of Fez Chouara in Morocco was taken in June 2019 (right).

Sumac (*Rhus coriaria L.*, *Anacardiaceae*) is found in Spain, southern Italy, Turkey, and the Middle Eastern countries [25,26,27,28]. There are more than 250 sumac species in the genus [28]. The fruits are reddish, thin-fleshed drupes covered in some hairs at maturity and form dense clusters at branch tips, sometimes called sumac bobs (Figure 3). All the red fruits of sumac are edible. Acids on hairs on the berries are used to make sumac-ade (a sweetened beverage prepared by soaking and rubbing the sumac drupes in cool water, which is followed by straining the obtained liquid through a tissue). The harvested fruits themselves can be used to make a spice by grinding and sieving. The hard bulb remains after grinding and the fine externally cleaned seeds can be used as an antioxidant-rich spice [26,27,29]. This has a lemon-like flavor and is often used in Levant cuisine [30,31]. Sumac fruits are used as dye plants in Morocco in “traditional tanneries of Fez Chouara” that treat and color the leather of animals (Figure 3). Sumac is also traditionally used in the Middle East, not only as a spice but also as a medicinal plant, e.g., to treat and prevent diabetes, constipated bowel complaints, febrile diseases or dysmenorrhea [26,32,33,34]. Consequently, the transfer of sumac into sumac PlantCrystals is a promising approach to foster the medicinal activity of this plant.

## 2. Results

The study consisted of two parts. The first part investigated if it is possible produce sumac PlantCrystals, i.e., to nanomill sumac bulk material into submicron sized sumac particles that can be freeze dried and resuspended whilst maintaining their nanoproperties (cf. Section 2.1.). The second part investigated the influence of milling and freeze drying on the bioactivity, i.e., the antioxidant capacity, of the sumac (cf. Section 2.2). A detailed description of the materials and methods used in this study is given in Section 4.

### 2.1. Production and Physicochemical Characterization Sumac PlantCrystals

**Figure 4:**
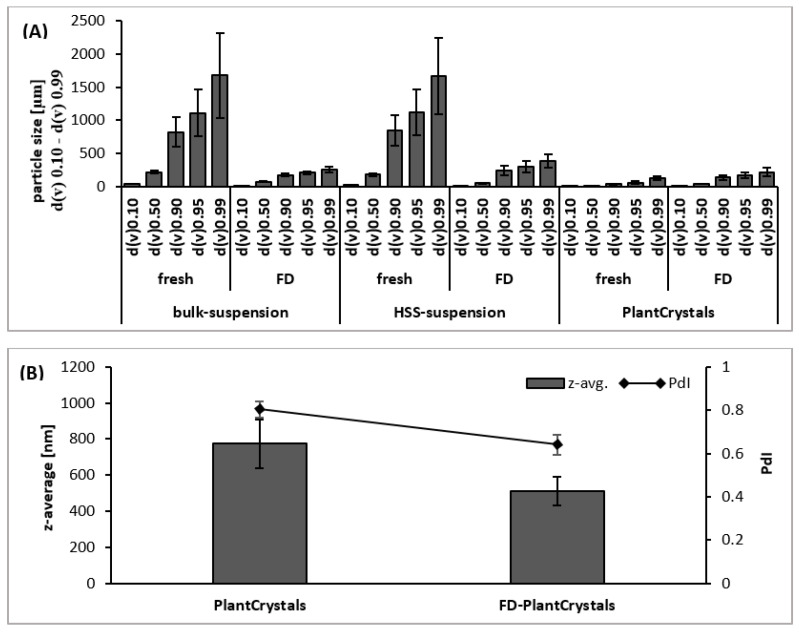
Size characterization of sumac formulations: (**A**) laser diffraction data showed particle size of sumac suspensions before and after freeze drying (FD): bulk, HSS and HPH (PlantCrystals) suspensions. (**B**) Dynamic light scattering (DLS) data show z-average and the polydispersity index (PdI) of the produced PlantCrystals of sumac before and after freeze drying (FD).

Sumac bulk material possessed a mean size of about 200 µm. HSS reduced the particle size to about 70 µm and HPH could further reduce it to about 10 µm (Figure 4A). DLS measurements also proved the presence of submicron sized particles (about 770 nm) (Figure 4B).

**Figure 5:**
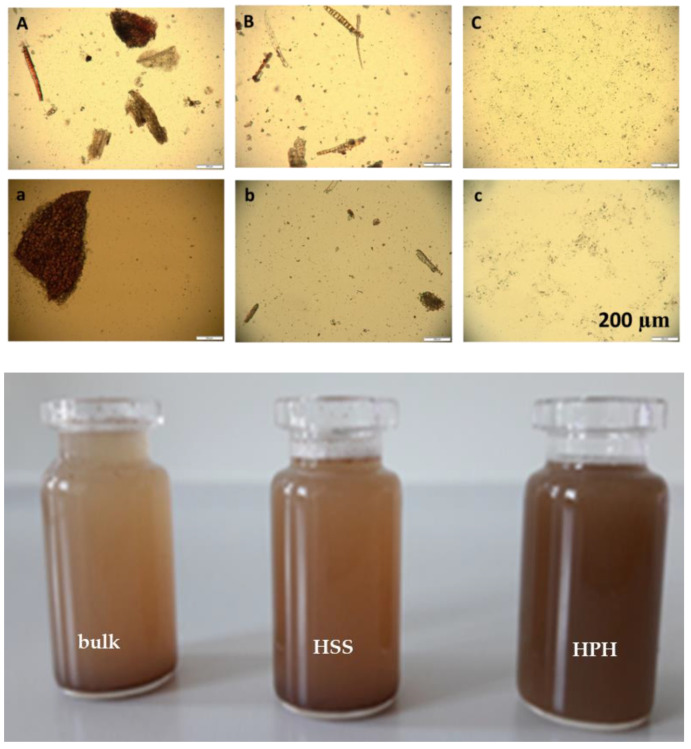
**upper:** Light microscopic images of sumac fresh suspensions (upper panel): (**A**) bulk-material and its (**B**) HSS-suspension and (**C**) PlantCrystals after HPH; sumac freeze dried (FD, lower panel) suspensions (**a**) FD-bulk-material and (**b**) FD-HSS-suspension (**c**) FD-PlantCrystals after redispersion (100-fold magnification and scale of 200 µm). **Lower:** Macroscopic images of the freshly produced suspensions of sumac, from left to right: bulk suspension, microsuspension after high speed stirring (HSS) and PlantCrystals after high pressure homogenization (HPH).

However, the polydispersity index was very high (about 0.8), indicating a very broad size distribution, which was also confirmed by the LD data (Figure 4). LD and DLS results also showed that the suspension after HPH is a mixture of micro- and nanosized particles. Hence, some larger particles remained during the HPH process. However, the number of these particles seems to be very small because light microscopy did not reveal such large particles for the HPH PlantCrystals (Figure 5-upper). Additionally, macroscopic observations confirmed a homogeneous distribution of the particles in the final PlantCrystals without any noticed sedimentation (Figure 5-lower).

Lyophilization (freeze-drying (FD)) converted the produced PlantCrystals into bridle cakes, which were easy to redisperse in water. Upon redispersion of the PlantCrystals, a slight increase in the particle size was detected (Figure 4A). Hence, some of the PlantCrystals formed aggregates that were not fully redispersed. However, DLS data confirmed that the main size population possesses a particle size well below 1µm (Figure 4B). Redispersion of the lyophilized bulk and HSS suspensions led to smaller particle sizes. In brief, lyophilization redispersion of PlantCrystals formed some aggregates, but it decreased the particle size of the bulk and micronized HSS suspensions.

The zeta-potential of the PlantCrystals was analyzed in water with a conductivity of 50 μS/cm to detect the charge on the PlantCrystals surface. Based on the zeta-potential measurements before and after lyophilization redispersion, the obtained values were in the range of −14 mV (±1) and −17 mV (±0.4), which classifies the PlantCrystals as mildly stable suspensions.

### 2.2. Extraction Efficacy and Antioxidant Capacity

#### 2.2.1. Total Polyphenol Content (TPC)

**Figure 6:**
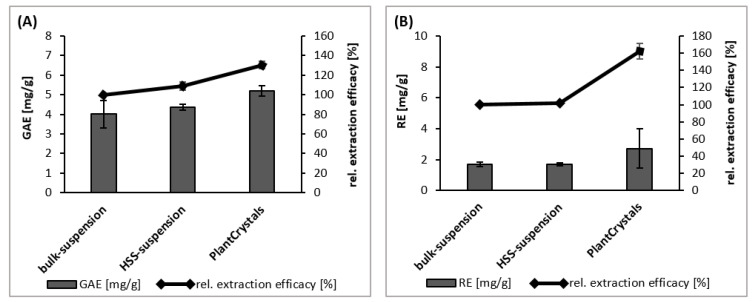
Determination of extraction efficacy, determined as total polyphenol content (**A**) and total flavonoid content (**B**) of sumac suspensions as bulk materials, micronized (HSS) and HPH-suspension (PlantCrystals). The polyphenol content is expressed in gallic acid equivalents (GAE), where gallic acid was used as benchmark reference. The flavonoid content is expressed as rutin equivalents (RE), where rutin was used as a as benchmark reference.

The TPC value of the suspension obtained after HPH (PlantCrystals) was significantly (*p* < 0.05) increased by 30% when compared to the bulk and micronized (HSS) materials (Figure 6A). Hence, the available (released) polyphenol content was higher from the PlantCrystals than from the other formulations due to the increased extraction efficacy of the nanosized sumac, caused by the more effective destruction of the plant cells by HPH.

#### 2.2.2. Total Flavonoid Content (TFC)

Almost no differences in the TFC values were found between bulk material and the micronized HSS-suspension (Figure 6B). However, the PlantCrystals obtained upon HPH possessed an almost 2-fold higher TFC value (Figure 6B). Hence, the diminution of the plant materials enabled a much better release of the flavonoids than the larger sized material. This difference was not significant.

#### 2.2.3. Electron Transfer (ET) Assays

Three different in vitro assays based on electron transfer were used to evaluate the antioxidant activity of sumac formulations produced in this study: scavenging activity on DPPH radicals, reductive power (FRAP) and (2,2′-azinobis(3-ethylbenzothiazoline-6-sulfonic acid)) assay (ABTS). All sumac suspensions showed a propensity to quench the free radicals.

##### DPPH^●^ (1,1-diphenyl-2-picrylhydrazyl) Assay

**Figure 7:**
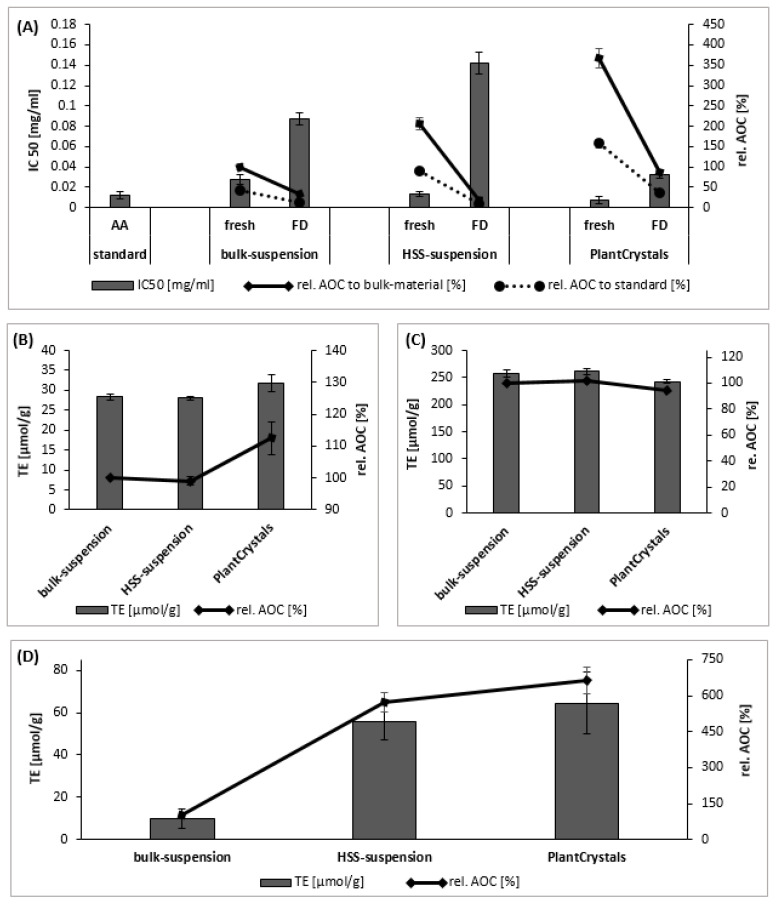
Determination of antioxidant capacity. (**A**) Antioxidant capacity using DPPH assay of sumac suspensions before (fresh) and after freeze drying (FD) as bulk materials, micronized (HSS) and HPH-suspension (PlantCrystals). Their IC50 values were compared to the IC50 of AA (ascorbic acid), which was used as standard. No significant difference was noticed between the fresh PlantCrystals after HPH and the used standard. (**B**) Antioxidant capacity using FRAP assay. (**C**) Antioxidant capacity using ABTS assay. (**D**) Antioxidant capacity using ORAC assay. FRAP, ABTS and ORAC values were expressed as µmol Trolox equivalent (TE) per g of the sample analyzed.

IC50 values represent the amount of active constituents needed to scavenge 50% of a given amount of free radicals, i.e., low IC50 values represent a high AOC [13,14]. The combination of HSS and HPH techniques could elevate the AOC of the unprocessed bulk-suspension of sumac in a significant way (*p* < 0.01) (Figure 7A). This indicated an about 3.6-fold increase in the AOC when compared to the bulk suspension (Figure 7A).

The rel. AOC represents the increase or decrease in AOC of plant samples when compared to ascorbic acid (standard) or the bulk suspension (Figure 7A). The fresh PlantCrystals from sumac fruits possessed a higher AOC than the ascorbic acid. The fresh bulk suspension possessed an AOC corresponding to about 40% of the AOC-value of the ascorbic acid. That was followed by an increase of about 90% and 160% for the fresh HSS and HPH sumac, respectively (Figure 7A). Interestingly, also the microsuspension (HSS-suspension) yielded such high AOC values. Additionally, the reason for the increased AOC values might be due to the destruction of plant cells, which causes an exhausting release of their active constituents. However, lyophilization redispersion process significantly increased the IC50 values, i.e., reduced the AOC of all the formulations produced in this study (i.e., bulk, HSS and HPH suspensions) (Figure 7A). Nonetheless, lyophilization redispersion, was an unavoidable step to store the formulations during the study period to perform the other assays, because otherwise the high water content would have caused microbial contamination and consequently destruction of the formulations during storage.

The decrease in the AOC of the lyophilized redispersed HSS-suspension is related to the nature of these active ingredients released, which are more hydrophilic and therefore can be degraded and oxidized rapidly upon redispersion in fresh water, which then results in a decrease in AOC when compared to bulk material or HPH PlantCrystals [13].

Despite the reduction in the AOC upon lyophilization, which was also demonstrated for the PlantCrystals [13], the FD-HPH PlantCrystals showed the best AOC in comparison to the other FD suspensions (Figure 7A).

##### FRAP (Ferric ion Reducing Antioxidant Power) Assay and ABTS (2,2′-azinobis(3-ethylbenzothiazoline-6-sulfonic acid)) Assay

Bulk-sumac had a similar value compared to the HSS-sumac (Figure 7B). However, HPH could increase the reducing ability of sumac antioxidants and showed an about a 13% increase in the FRAP value of bulk-sumac. Thus, the combination of HSS and HPH could significantly (*p* < 0.05) increase the extraction efficiency of sumac antioxidants and led to an elevated AOC (Figure 7B).

The ABTS assay values for sumac formulations ranged from 240 to 260 µmol TE/g and indicated a good AOC of the produced sumac formulations (Figure 7C). Despite the slight decrease in the ABTS value upon HPH, the final sumac PlantCrystals showed almost similar activity. Thus, almost no or only slight changes were noticed in the sumac evaluated formulations in the ABTS assay. The rel. AOC _ABTS_ was 102% and 94% for the HSS suspension and HPH PlantCrystals compared to the bulk sumac suspension. The values of DPPH and FRAP assays showed higher antioxidant activity than ABTS for PlantCrystals (compared to the bulk and HSS suspensions). It is also reported in other studies (for different plant extracts) that ABTS assay showed lower sensitivity than the other ET methods and that the values of the three methods can be varied [35]. Thus, it is important to mention that antioxidant compounds can respond in a different way to different radicals or oxidants, meaning that the use of only one AOC assay can lead to misleading results. Therefore, to obtain detailed information on the AOC of plant material, different AOC assays must be applied and the results obtained need to be compared to each other (AOC test battery).

Measuring antioxidant capacity by methods such as DPPH, ABTS, and FRAP is characterized by the reduction of free exogenous radicals, such as DPPH^•^, ABTS+^•^ and the TPTZ–Fe (III) complex, through the transfer of electrons from molecules [36]. Thus, their reaction mechanisms are similar, which partly explains the similarity of the results of these tests. Additionally, in both ABTS and FRAP assays, the redox potential of the antioxidant phytochemicals in the tested formulations is important. The redox potential of Fe(II)/(III) and of the redox couple ABTS/ABTS+^•^ are comparable with values of 0.70 and 0.68 V, respectively [10,36]. Thus, these compounds should react in a similar way in both ABTS and FRAP assays, which should lead to a good correlation between their results. This is also clear in our study that almost no or slight changes were noticed between the different formulations (bulk, HSS and HPH) in FRAP and ABTS assays.

#### 2.2.4. Hydrogen Atom Transfer (HAT) Assays

##### ORAC (Oxygen Radical Absorbance Capacity) Assay

ORAC assay was used to gain a better understanding of the total AOC of the produced PlantCrystals from sumac. The ORAC method is characterized by capturing the endogenous radicals that act on oxidized targets and thus is a meaningful tool to express the antioxidant capacity (AOC) of a formulation [13]. In addition, the ORAC assay is considered to be most suitable to assess hydrophilic and lipophilic antioxidants at the same time [37]. The ORAC values after micronization (HSS) and after nanosziation (HPH) were 56 and 65 µmol TE/g, respectively.

The AOC-values obtained by the ORAC assay show a similar trend as the values obtained from the DPPH assay and demonstrate a pronounced increase (*p* < 0.001) in the AOC for the PlantCrystals upon HPH, which finally results in a 6.4-fold and 5.6-fold higher AOC when compared to the AOC of the bulk and HSS suspensions, respectively (Figure 7D).

## 3. Discussion

HPH was chosen in this study as a well-established and eco-friendly technique to investigate its effect on the elevated release of antioxidants from sumac fruits due to the ability of HPH to disrupt plant cells. Diminution of particles by HPH is based on Bernoulli law [38]. In brief, the plant bulk-suspension is forced to pass through a small orifice that simultaneously leads to a reduction of the static pressure (in the homogenizer gap) below the boiling pressure. This, in turn, leads to the boiling of the fluid and the formation of gas bubbles. Afterwards, the suspension leaves the homogenization gap and normal air pressure conditions are achieved again. This causes the air bubbles to implore immediately. The effect is called cavitation and creates shock waves which then lead to the destruction of particles being suspended in the water phase. Additional forces that occur during HPH and further promote size reduction of the particles include impact and shear forces. As demonstrated, cavitation also causes a reduction in plant particles size and breaks the plant cell walls. Thus, achieving a pronounced release of plant active ingredients [38]. Adjusting the temperature during the homogenization cycles was an unavoidable step to minimize the possible thermal damage of the materials due to the HPH. This thermal damage can be a result of the friction heat caused due to the high fluid velocity, which elevates the temperature of the suspension about 2–2.5 °C/100 bar [39].

Sumac PlantCrystals were successfully produced by using HPH. LD data showed that the PlantCrystals possessed a particle size in the submicron range with some remaining larger sized microparticles. However, all particles were well below 10 µm (the approximate plant cell size), indicating that all plant cells were successfully destroyed. The particle distribution of the sumac PlantCrystals was broad. The reason is the presence of a mixture of micronized and nanosized plant particles and the presence of manifold and diverse sumac fruit fragments (for example parts of hairs, pulp, kernels, etc.) that responded differently to the milling forces applied, thus leading to different particle sizes. Freeze-dried and resuspended PlantCrystals possessed slight differences in sizes, indicating that freeze-drying did not significantly alter the size of the formulations. It led to smaller particle sizes for the unprocessed bulk-suspension and HSS-suspension but resulted in larger particles for the HPH PlantCrystals. This might be a result of slight agglomeration of some individual, nanosized PlantCrystals.

The zeta-potential was analyzed to predict the physical stability of the PlantCrystals. All the produced PlantCrystals (before and after lyophilization redispersion) had values between −14 and −17 mV in the final formulation, indicating a decent stability due to steric stabilization of the nonionic surfactant used.

HPH resulted in a comprehensive destruction of the plant cells of sumac (particle size < 10 µm, the size of a plant cell). The destruction of the plant cells resulted—as expected—in an increased extraction of sumac phytochemicals, i.e., polyphenols and flavonoids. An increase in extraction efficacy and with this an increase in the antioxidant capacity of sumac was already seen after HSS (+8.8%). However, a more pronounced increase (+30%) was noticed upon HPH. The observed highest TPC value of the PlantCrystals obtained after HPH agreed with the results obtained by Saldo et al. [18] and Schilling et al. [40] (for juices from different materials). This can be related to the release of these molecules upon breaking the plant cells but also to the preservation of the phenolic compounds, due to an inactivation of polyphenol oxidase enzymes by HPH [18,39].

Despite an increase in TPC, HPH also increased the total flavonoid content (TFC). Here, the effect of HSS was neglectable (+1%) but more pronounced for the HPH (+62%) when compared to the TPC. Data therefore show again that the PlantCrystal-technology is especially suitable for an improved extraction of more lipophilic compounds. This is because HPH interrupts not only the plant cells but also the lipophilic membranes and cell compartments in which these compounds are stored. Thus, HPH but not HSS fosters the release of these compounds. Sumac contains > 60 flavonoid derivatives like quercitrin, quercetin, rhamnetin, isovitexin, hesperidin, rutin, myricitrin, apiin, apigenin and kaempferol with manifold pharmacological properties [34,41,42,43]. The release of these compounds from the plant cells upon HPH provides higher amounts of dissolved flavonoids and thus can increase their bioavailability by increasing their availability for absorption after fermentation by the gut microflora [41,44,45,46,47]. In contrast, the flavonoids in the unprocessed bulk plant materials are less accessible (in comparison to the nanosized plant materials) to the action of the intestinal microbial enzymes and thus the use of unprocessed plant material will cause a lower bioavailability of these compounds [48]. These increased flavonoid contents after HPH agreed with our previous findings (for other plants) and with the results published by Velázquez-Estrada et al. (for the orange juice) [17].

The accelerated extraction of polyphenols and flavonoids upon milling increased the bioactivity of sumac, which was measured as antioxidant capacity. The application of HSS led to reduced sizes in the upper micrometer level. This leads to a pronounced release of hydrophilic antioxidants. As already mentioned above, HPH technique is more effective to disrupt the plant cells and cell organelles that host the hydrophobic antioxidants and this results in the release of more hydrophobic antioxidants [13]. Consequently, the smaller sizes of the PlantCrystals led to a higher release of these antioxidants.

A significant decrease in the AOC of the freeze-dried and redispersed (in water) formulations was noticed. This might be due to the oxygen contained in the freshwater, which can oxidize the antioxidants—upon the freeze drying and redispersion process—thus the more hydrophilic the antioxidants, the more they are prone to the oxidation process. Additionally, the hydrophilic antioxidants in the lyophilized samples get into contact with the redispersion medium quicker. Since, the HSS suspension contains more hydrophilic antioxidants than the bulk material [13], a more pronounced decrease in its AOC upon redispersion than the bulk suspension was observed.

In contrast, the PlantCrystals contain both hydrophilic and lipophilic antioxidants and the hydrophobic antioxidants protect the hydrophilic antioxidants from the oxidation to some extent [13]. Therefore, the all over decrease in AOC upon freezing and redispersion is less for the PlantCrystals when compared to the HSS formulation.

Previous studies on sumac extracts’ antioxidant potency demonstrated the high antioxidant activity of sumac by using different assays [26,29,32,41]. The high AOC of sumac has been mainly ascribed to gallic acid, ascorbic acid, hesperidin, rutin, apigenin, kaempferol, anthocyanins, hydrolysable tannins, carotenoids, terpenoids and other phenolic compounds [25,28,32,42]. The results obtained in this study are in line with these previous findings. The DPPH assay revealed already very low IC50 values for the bulk sumac. HSS and HPH could further decrease the IC50 values and resulted in lower IC50 values than the control (ascorbic acid). The extremely high AOC of sumac PlantCrystals after HPH can be associated with the destruction of the cellular structure and thus to a higher release of the lipophilic antioxidants from the plant compartments. Hence, HPH increased the availability of insoluble phytochemicals upon HPH, such as carotenoids, hesperidin or kaempferol, and thus increased their detectable AOC values in the sumac-PlantCrystals [17,25,28,32,39,42]. For ABTS and FRAP assays, almost no or only slight changes in the AOC were noticed upon the HPH applied on sumac fruits. These two assays are characterized by the reduction of the given free radicals, through the transfer of electrons from molecules [10,36]. The redox potential of Fe(II)/(III) and of the redox couple ABTS/ABTS+^•^ are comparable, which explains their results [10,36].

AOC of sumac is ascribed to contain both soluble and insoluble phytochemicals [25]. Therefore, the total AOC of the formulation is related to the soluble and insoluble active compounds [49]. Despite the previous assays, an additional AOC assay with a different mechanism is necessary to gain a more detailed view of the AOC of sumac PlantCrystals. Therefore, the ORAC assay was also performed. The AOC-values obtained by ORAC assay show a similar trend as the values obtained from the other assays performed in this study and confirm a significant increase in AOC for the PlantCrystals upon nanomilling.

The results therefore prove that the PlantCrystal extraction method is a two-step process. In the first step, the plant material is milled to sizes in the upper micrometer level. This results in a pronounced release of hydrophilic antioxidants, which were more prone to oxidation process after lyophilization redispersion. In the second step, nanomilling decreases the size of the plant material to sizes < 1µm. This leads to the destruction of all plant cells and cell organelles causing an exhausting release of their active constituents. The antioxidative molecules released in the second step are more lipophilic, due to the rupture of the lipophilic cellular compartments that contain these compounds [13].

The pronounced release of antioxidants upon HPH increases the antioxidant capacity of sumac by a factor 6.5 (ORAC-values) and thus provides evidence that HPH is a versatile process to improve the efficacy of sumac. However, besides a high antioxidant capacity, sumac possesses various other pharmacological properties, like antifungal, antibacterial, antiseptic, non-mutagenic, fever-reducing, DNA protective, anti-ischemic, hypouricemic, hypoglycemic, and hepatoprotective properties, which have already been exploited for a long time in folk medicine and traditional Arabic Palestinian herbal medicine [43,50]. Therefore, based on the outcome of this study, it can be expected that the transfer of sumac into sumac PlantCrystals increases not only the AOC of sumac but also its other biological properties. Studies that investigate the influence of HSS and HPH of sumac on its specific pharmacological properties in detail are now needed to prove this theory and to define most suitable production parameters (homogenization pressure, number of homogenization cycles, etc.) to obtain high extraction yields. Further studies are also required to reduce the degradation of the chemically labile hydrophilic antioxidants during the production process, which can be probably achieved by producing the PlantCrystals under a protective gas atmosphere.

## 4. Materials and Methods

### 4.1. Materials

Dried and ground sumac fruits were bought from a local market in Nablus, Palestine. Polysorbate 80 (Tween 80^®^, Sigma-Aldrich, Darmstadt, Germany) was used as surfactant. Purified water was used as dispersion medium and was obtained from a PURELAB^®^ Flex 2 water purification system (ELGA Labwater, Veolia Water Technologies Deutschland GmbH, Celle, Germany).

### 4.2. Methods

#### 4.2.1. Production of Sumac PlantCrystals

**Figure 8:**
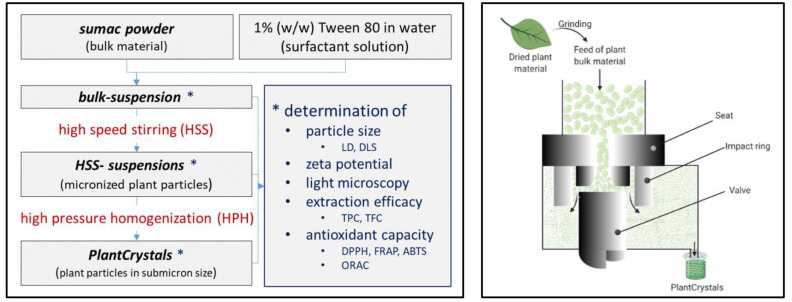
Scheme of the experimental setup (**left**) and principle of high pressure homogenization to produce PlantCrystals (**right**, modified after [52]). LD = laser diffraction, DLS = dynamic light scattering, TPC = total polyphenol content, TFC = total flavonoid content, DPPH = 1,1-diphenyl-2-picrylhydrazyl assay, FRAP = ferric ion reducing antioxidant power assay, ABTS = 2,2′-azinobis(3-ethylbenzothiazoline-6-sulfonic acid) assay, ORAC = oxygen radical absorbance capacity assay.

Sumac bulk material was further dry-grinded to obtain a fine powder by using mortar and pestle, a hand blender (Elta Lizenz GmbH, Oststeinbeck, Germany) and an AR1105 electrical grinder (Moulinex, Grenoble, France). In the next step, 1% (*w*/*w*) of the so obtained sumac bulk powder was suspended in a surfactant solution that contained 1% (*w*/*w*) Tween 80 as surfactant to obtain the sumac bulk suspension. The surfactant solution was used to physically stabilize the produced sumac particles and prevent their agglomeration. Subsequently, the bulk-suspension was premilled using high speed stirring (HSS) by a rotor-stator mixer (Ultra Turrax T25, IKA, Königswinter, Germany) employing different rotation speeds to obtain what was called a HSS or microsuspension. The microsuspension obtained was subjected to the nanomilling process using high-pressure homogenization (HPH) with a LAB 40 piston gap homogenizer in discontinuous mode with a batch size of 40 mL (GEA Soavi, Lübeck, Germany) at 1500 bar for up to 30 cycles to yield the PlantCrystals (Figure 8) [13]. A water bath was used to avoid the possible thermal damage of the sumac phytochemicals during HPH with a temperature set to 5 °C [51]. The suspensions obtained were immediately analyzed regarding size, zeta potential and antioxidant capacity. Subsequently, they were freeze-dried to enable long-time storage without microbiol degradation.

#### 4.2.2. Freeze Drying of Sumac PlantCrystals

PlantCrystals are prone to microbiological contamination. To improve the suspensions’ shelf life and to use them in the following experiments, all the formulations were lyophilized directly after production. Lyophilization was performed using an Alpha 1-4 LSC lyophilizer (Martin Christ Gefriertrocknungsanlagen GmbH, Osterode am Harz, Germany). The samples were frozen at (−80 °C) overnight, then main dried (−50 °C, 0.120 mbar) for 48 h and the final drying (25 °C, 1 mbar) was performed for 24 h. Mannitol 20% *w/v* was used as a cryoprotectant [13]. Lyophilized samples were homogenized using mortar and pestle and then redispersed in purified water directly before analysis to obtain 1% (*w*/*w*) plant material.

#### 4.2.3. Physicochemical Characterization Sumac PlantCrystals

In-process and at the end of the production particle sizes and degree of agglomeration of bulk material, HSS and HPH suspensions were characterized using a combination of three methods of particle size characterization on the same day of the production and/or redispersion. This includes light microscopy equipped with SC50 CMOS color camera (Olympus soft imaging solutions GmbH, Münster, Germany), dynamic and static laser light scattering (Zetasizer Nano ZS and Mastersizer 3000, Malvern-Panalytical, Kassel, Germany). Dynamic light scattering (DLS) results show the hydrodynamic diameter (z-average) and the polydispersity index (PdI); as an indication of the width of the size distribution. Static laser light scattering (SLS), or what is also called laser diffraction (LD), was performed to detect the possible large particles that can remain after HPH. Mie-theory with optical parameters set to 1.45 (real refractive index) and 0.01 (imaginary refractive index) was used for the LD analysis. Sonication was avoided during the measurements to evade any resulted breaking of the possible agglomerates [53].

Zeta-potential (ZP) was measured at 20 °C by using Zetasizer Nano ZS (Malvern-Panalytical, Kassel, Germany). To measure the ZP, the electrophoretic mobility was determined with Laser-Doppler-anemometry (LDA). Then Malvern Zetasizer software converted the electrophoretic mobility into the ZP by using Helmholtz–Smoluchowski equation. The measurements were performed in conductivity adjusted purified water (50 μS/cm). The analysis was performed in triplicates and shown as an average ± the mean standard deviation (SD).

#### 4.2.4. Determination of Extraction Efficacy and Antioxidant Capacity

The extraction efficacy and the AOC were assessed with a battery of different test methods and included analysis of the Total Polyphenol Content (TPC), the Total Flavonoid Content (TFC) and the determination of the AOC by different AOC assays (DPPH, FRAP, ABTS and ORAC assay). Detailed information on the assays performed are described below.

##### Total Polyphenol Content (TPC)

TPC was measured by the Folin–Ciocalteu method [10,54,55]. The reaction mixture contained purified water, sample or standard, 20% sodium carbonate (Merck Chemicals, Darmstadt, Germany), and 2N Folin–Ciocalteu reagent (Merck Chemicals, Darmstadt, Germany) at a ratio of 7.5:1:1:0.5. After one hour at room temperature and with protection from light, the absorbance was measured at 760 nm using a UV/Vis spectrophotometer (BioTek Instruments, Winooski, VT, USA). Then results were expressed in mg gallic acid (Sigma-Aldrich Chemical Co., Louis, MO, USA) equivalents per gram of sample (mg GAE/g) based on a calibration curve with gallic acid (10–100 µg/mL). In addition, results were expressed as relative extraction efficacy (%) of these phenolic compounds in comparison to the nonprocessed bulk material.

##### Total Flavonoid Content (TFC)

TFC was determined using a method being based on the interaction of flavonoids with AlCl_3_ (Merck Chemicals, Darmstadt, Germany) that leads to the formation of a complex that can be determined via a UV/Vis spectrophotometer (BioTek Instruments, Winooski, VT, USA) at 420 nm [54]. The TFC values are expressed as mg rutin (Cayman Chemical Co., MI, USA) equivalents (RE) per gram of suspension based on a calibration curve with rutin (10–100 µg/mL). In addition, results were expressed as relative extraction efficacy (%) and compared to the nonprocessed bulk material.

##### Electron Transfer (ET) Assays

DPPH^●^ (1,1-diphenyl-2-picrylhydrazyl) Assay

The DPPH^●^ (1,1-diphenyl-2-picrylhydrazyl, Sigma–Aldrich Chemie GmbH, Steinheim am Albuch, Germany) assay according to the method proposed by Sharma and Bhat [13,15,55,56] was used to analyze the antioxidant capacity of the fresh and lyophilized sumac PlantCrystals. Results were compared to the AOC of the HSS-suspension and bulk materials, and to ascorbic acid that was used as benchmark control. The test was performed in a 96-well plate. Initially, a 0.2 mM DPPH solution was prepared in methanol. Afterward, dilution of each sample was done using distilled water and finally 100 µL of DPPH solution was added. Methanol in addition to distilled water were used as blanks. That was followed by incubating the plates in the dark for 30 min to allow the reaction to occur. In the next step, the absorbance was measured by a Multi-plate UV/Vis spectrophotometer at a wavelength of 517 nm by using Multiskan GO (Thermo Scientific, Dreieich, Germany). The assay was performed in triplicate and then the percentage of radical scavenging activity (RSA) values were calculated from the following equation:

$$RSA\;\left(\%\right)=\left(\frac{absorbance_{DPPH}-absorbance_{sample}}{absorbance_{DPPH}}\right)\times 100$$

DPPH results were determined by the IC 50% and as a relative antioxidant capacity compared to the nonprocessed bulk sumac.

##### FRAP (Ferric ion Reducing Antioxidant Power) Assay

This method evaluates the capacity of the antioxidants in the samples by reducing the ferric ion (Fe^3+^) in an acidic medium in the presence of TPTZ (2,4,6-Tris(2-pyridyl)-s-triazine) to form the ferrous form (Fe^2+^). This reaction leads to a reduced TPTZ–Fe (III) complex with blue color, measured at 593 nm. The working solution contained 300 mM acetate buffer (pH 3.6), a 40 mM TPTZ solution (Sigma-Aldrich Chemical Co., Louis, MO, USA), and a 20 mM FeCl_3_·6H_2_O solution (Merck Chemicals, Darmstadt, Germany) in water at a 10:1:1 ratio. Suspensions and the working FRAP solution were mixed at a 1:25 ratio for 10 min at 37 °C in a dark place. The absorbance was taken at 593 nm using a UV/Vis spectrophotometer (BioTek Instruments, Winooski, VT, USA) [10]. A calibration curve with Trolox (Sigma-Aldrich Chemical Co., Louis, MO, USA) was used. The results are expressed in µmol Trolox equivalents per gram of suspension and relative antioxidant capacity compared to the nonprocessed bulk material.

##### ABTS (2,2′-azinobis(3-ethylbenzothiazoline-6-sulfonic acid)) Assay

In the ABTS assay, the greenish-blue stable radical cation ABTS^•+^ (2,2′-azinobis(3-ethylbenzothiazoline-6-sulfonate) is produced by oxidation and has an absorbance maximum at 734 nm. The absorbance was measured using a UV/Vis spectrophotometer (BioTek Instruments, Winooski, VT, USA). ABTS^•+^ was generated by the reaction of 7 mM ABTS (Sigma-Aldrich Chemical Co., Louis, MO, USA) and 2.45 mM potassium persulfate (Merck Chemicals, Darmstadt, Germany) in PBS (pH: 7.4) in a dark place at room temperature for 16 h. ABTS values are expressed in µmol Trolox (Sigma-Aldrich Chemical Co., Louis, MO, USA) equivalents per gram of sample (µmol TE/g). The calculations were based on a Trolox calibration curve vs. the inhibition percent of the radical ABTS^•+^. The redispersed samples were added after ABTS^•+^ was generated [10]. The data were also expressed as relative antioxidant capacity in comparison to the nonprocessed bulk material.

##### Hydrogen Atom Transfer (HAT) Assays

The ORAC (oxygen radical absorbance capacity) assay was assessed. For this AAPH (2,2′-azobis(2-methylpropionamidine) dihydrochloride, Merck Chemicals, Darmstadt, Germany) was used as a peroxyl radical generator, fluorescein (Merck Chemicals, Darmstadt, Germany) as fluorescent and Trolox (6-hydroxy-2,5,7,8tetramethylchromane-2-carboxylic acid, Merck Chemicals, Darmstadt, Germany) as a standard. The fluorescein intensity was measured every minute for 2 h at excitation and emission wavelengths of 485 and 520 nm, respectively. ORAC values were expressed as µmol Trolox equivalents per gram of suspension [10,57,58].

#### 4.2.5. Statistical Analysis

All results were expressed as mean ± SD. All statistical analyses were performed using GraphPad Prism 5 (GraphPad Software Inc., San Diego, CA, USA). Analysis of variance and Tukey’s multiple comparison tests were performed to evaluate significant (*p* < 0.05) differences between bulk-materials, HSS and HPH PlantCrystals.

## 5. Conclusions

Sumac fruit was successfully rendered into sumac PlantCrystals by using a combination of HSS and HPH. The obtained sumac PlantCrystals possessed a size < 1 µm and thus contained no intact plant cells anymore. The destruction of the cells enabled a more exhaustive release of antioxidative plant compounds and thus could increase the total AOC of sumac to > 650% when compared to sumac bulk material. Thus, proving that the PlantCrystal-technology can be used to increase the biological activity of sumac fruit. Like previous studies, also this study confirmed that HSS as a pretreatment step mainly causes the release of hydrophilic compounds, whereas HPH—due to the destruction of the plant membranes—also enables the release of hydrophobic compounds. Freeze drying of the sumac PlantCrystals was performed to improve the microbial stability. The lyophilization process was not affecting the physical stability of the PlantCrystals but caused a significant decrease in the AOC, where hydrophilic antioxidants were more affected by degradation than the more lipophilic compounds. Future studies are now required to understand the phenomenon in detail and to optimize the lyophilization process and/or to develop alternative processes that maintain microbial, physical, and chemical stability of the PlantCrystals at the same time.

Based on the results obtained it can be concluded that the PlantCrystal-technology is a feasible approach to improve the efficacy of sumac fruit, which enables the production of highly effective and eco-friendly plant extracts from sumac fruits that can be used for the production of natural personal-care products and cosmetics with high antioxidative capacity. Further research is needed to investigate if sumac PlantCrystals are also useful for the production of herbal drug products.

## Data Availability

All data generated or analyzed during this study are included in this published article.
